# Supplementary material for: Comparative multiomics analysis of cell physiological state after culture in a basket bioreactor
Source: Sci Rep. 2022 Nov 23;12:20161. doi: 10.1038/s41598-022-24687-4 (PMC9686226; doi:10.1038/s41598-022-24687-4)
Supplement: Supplementary file 1 — Supplementary Information 1. [file 41598_2022_24687_MOESM1_ESM.zip › raw data/Proteomic raw data/2.func.anno/2.4.IPR/IPR.anno.pdf]

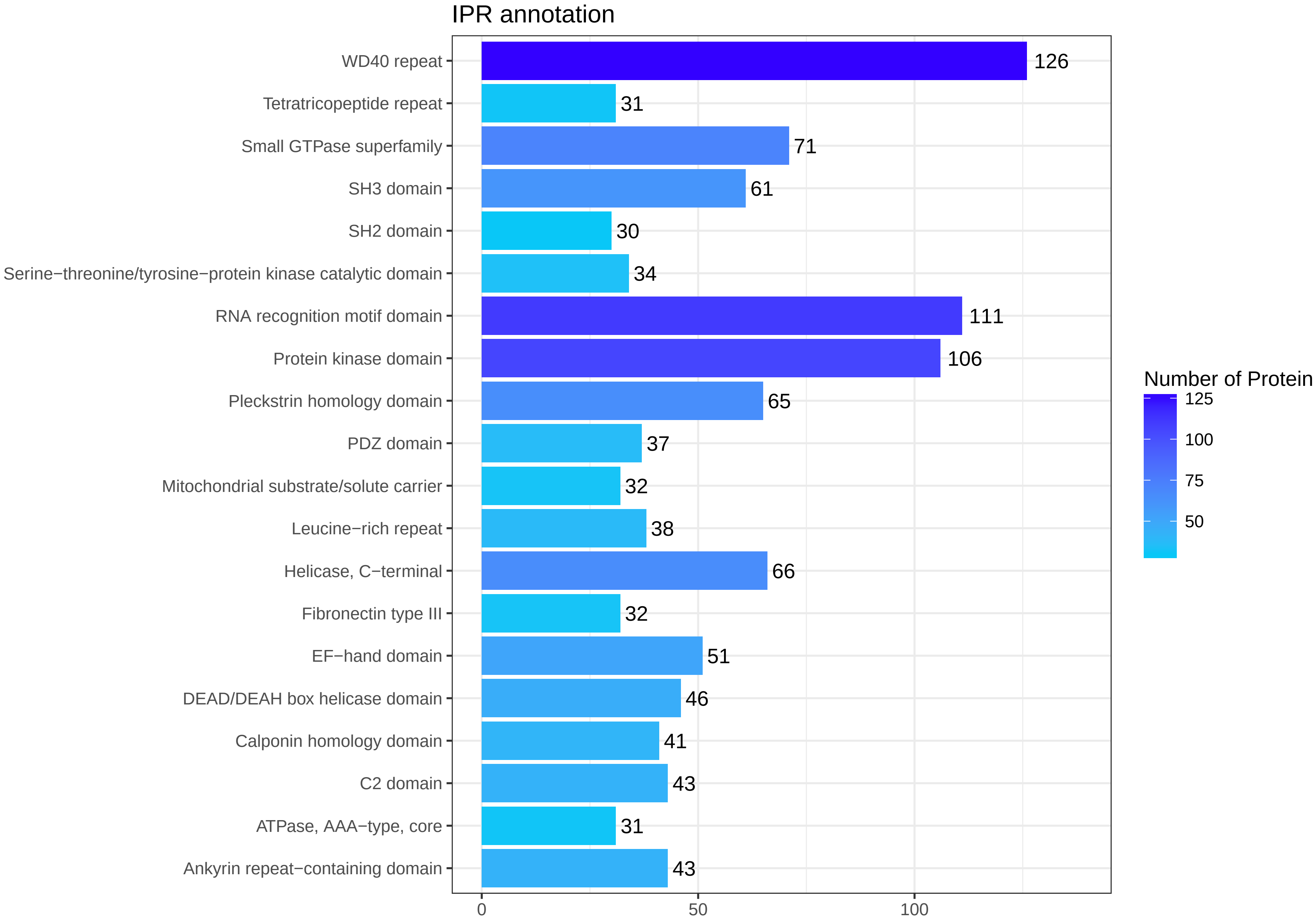

IPR annotation
WD40 repeat
Tetratricopeptide repeat
Small GTPase superfamily
SH3 domain
SH2 domain
Serine−threonine/tyrosine−protein kinase catalytic domain
RNA recognition motif domain
Protein kinase domain
Pleckstrin homology domain
PDZ domain
Mitochondrial substrate/solute carrier
Leucine−rich repeat
Helicase, C−terminal
Fibronectin type III
EF−hand domain
DEAD/DEAH box helicase domain
Calponin homology domain
C2 domain
ATPase, AAA−type, core
Ankyrin repeat−containing domain
126
31
71
61
30
34
111
106
65
37
32
38
66
32
51
46
41
43
31
43
0
50
100
Number of Protein
125
100
75
50
